# Dynamic Computational Model of Symptomatic Bacteremia to Inform Bacterial Separation Treatment Requirements

**DOI:** 10.1371/journal.pone.0163167

**Published:** 2016-09-22

**Authors:** Sinead E. Miller, Charleson S. Bell, Mark S. McClain, Timothy L. Cover, Todd D. Giorgio

**Affiliations:** 1 Department of Biomedical Engineering, Vanderbilt University, Nashville, Tennessee, United States of America; 2 Department of Medicine, Division of Infectious Disease, Vanderbilt University Medical Center, Nashville, Tennessee, United States of America; 3 Veterans Affairs Tennessee Valley Healthcare System, Nashville, Tennessee, United States of America; Cornell University, UNITED STATES

## Abstract

The rise of multi-drug resistance has decreased the effectiveness of antibiotics, which has led to increased mortality rates associated with symptomatic bacteremia, or bacterial sepsis. To combat decreasing antibiotic effectiveness, extracorporeal bacterial separation approaches have been proposed to capture and separate bacteria from blood. However, bacteremia is dynamic and involves host-pathogen interactions across various anatomical sites. We developed a mathematical model that quantitatively describes the kinetics of pathogenesis and progression of symptomatic bacteremia under various conditions, including bacterial separation therapy, to better understand disease mechanisms and quantitatively assess the biological impact of bacterial separation therapy. Model validity was tested against experimental data from published studies. This is the first multi-compartment model of symptomatic bacteremia in mammals that includes extracorporeal bacterial separation and antibiotic treatment, separately and in combination. The addition of an extracorporeal bacterial separation circuit reduced the predicted time of total bacteria clearance from the blood of an immunocompromised rodent by 49%, compared to antibiotic treatment alone. Implementation of bacterial separation therapy resulted in predicted multi-drug resistant bacterial clearance from the blood of a human in 97% less time than antibiotic treatment alone. The model also proposes a quantitative correlation between time-dependent bacterial load among tissues and bacteremia severity, analogous to the well-known ‘area under the curve’ for characterization of drug efficacy. The engineering-based mathematical model developed may be useful for informing the design of extracorporeal bacterial separation devices. This work enables the quantitative identification of the characteristics required of an extracorporeal bacteria separation device to provide biological benefit. These devices will potentially decrease the bacterial load in blood. Additionally, the devices may achieve bacterial separation rates that allow consequent acceleration of bacterial clearance in other tissues, inhibiting the progression of symptomatic bacteremia, including multi-drug resistant variations.

## Introduction

Symptomatic bacteremia is a common cause of severe sepsis[1]. In the United States, Gram-negative bacteria cause approximately 70% of hospital acquired infections in intensive care units[2], while up to half of all bloodstream infections are caused by Gram-negative bacilli[3]. Infections caused by Gram-negative bacteria are of particular concern because these organisms are highly efficient at acquiring and up-regulating various mechanisms that promote multi-drug resistance (MDR)[4]. Global concern revolves around the ever-increasing number of infections caused by MDR Gram-negative bacteria, in particular *Acinetobacter baumannii* and *Klebsiella pneumoniae*[5].

*A*. *baumannii* and *K*. *pneumoniae* are both Gram-negative bacilli with MDR phenotypes. These bacteria are commonly found in the hospital setting and reside in internal parts of the human body[6,7]. One of the strongest risk factors for developing a Gram-negative bloodstream infection is a compromised immune system. More specifically, those suffering from neutropenia, or lack of neutrophils in the blood, run a significantly higher risk for developing a bloodstream infection than healthy individuals[8,9]. *A*. *baumannii* and *K*. *pneumoniae* infections can lead to multiple consequences, such as widespread inflammation, blood clotting, multiple organ failure, pneumonia, septic shock, and death. The crude mortality rate associated with an *A*. *baumannii* or *K*. *pneumoniae* bloodstream infection in an immunocompromised patient can exceed 70%[8,10,11].

Treatment of a bloodstream infection requires eradication of bacteria from the blood, but also from other tissues, including the source of infection. The clearance of bacteria by the host immune response is similar to the pharmacokinetic clearance of drugs. Therefore, the clearance of bacteria from the host may be interpreted using a multi-compartment model with each compartment representing a tissue into which bacteria may enter and exit, replicate, or be eliminated by treatment. Bacteria can be transferred among tissues and eventually be eliminated through an immune response, therapeutic treatment, or a combination of both. Unlike drugs, bacteria proliferate prior to host recognition, and bacteria continue to proliferate in the case of an unsuccessful host defense.

Bacterial clearance from the bloodstream in a physiologically based pharmacokinetic (PBPK) model was first described in 1983 by Cheewatrakoolpong et al.[12]. The group analyzed the kinetics of bacterial clearance from the blood and mesenteric lymph nodes of mice using a two-compartment computational model. More recently, Kang et al. developed a mathematical model that predicts bacterial clearance solely from the bloodstream[13]. In the Kang et al. model, the group incorporated the effects of an extracorporeal bacterial separation device that continuously removes bacteria from flowing blood using a magnetic nanoparticle-based separation technique. The mathematical model successfully predicted the optimal magnetic nanoparticle sizes required for removal of bacteria from whole blood. This model enabled predictions of particle–pathogen collision and magnetophoresis rates, which allowed for determination of how these factors influence magnetic pathogen separation from blood under flow using a microfluidic magnetic separation device. The biological impact of bacterial separation in the host, however, was not incorporated into this model.

The work by Kang et al. can be expanded into a five-compartment model that accounts for physical and immunological interactions, bacterial net growth, transport among tissues (lungs, spleen, liver, blood), antibiotic treatment, and extracorporeal removal of bacteria from the blood. This type of expanded, dynamic model can be used to characterize time-dependent, tissue-specific bacterial load in symptomatic bacteremia. Also, such a model could be used to characterize the biological impact of bacterial separation from the blood, and, potentially, predict the utility as a therapeutic treatment option in conjunction with antibiotic treatment. Consequently, we developed a pharmacokinetic model of *A*. *baumannii* and *K*. *pneumoniae* symptomatic bacteremia in order to provide a quantitative and flexible framework useful for both experimental and analytical work in this area.

A five-compartment dynamic model, consisting of five first-order homogenous ordinary differential equations, was developed to study the biodistribution of *A*. *baumannii* and *K*. *pneumoniae* during symptomatic bacteremia in mammals. This pharmacokinetic model was used to assess the combination of broad-spectrum colistin antibiotic treatment and extracorporeal bacteria separation from the blood on the overall time-dependent bacterial burden in infected living systems. Colistin, a naturally occurring cationic decapeptide isolated from *Paenibacillus polymyxa* var. *colistinus*[14], is a potent broad-spectrum antimicrobial. This antibiotic is commonly used for the treatment of challenging Gram-negative pathogens[15], including *A*. *baumannii*.

The time course of a bacterial infection within important tissues of immunologically normal rodents was described using the mathematical model. The same measures were then used to examine the impact of suppressed immunity. The efficacy of antibiotic administration in immunosuppressed rodents exposed to a bacterial challenge was also analyzed. Finally, the potential benefit of extracorporeal bacterial isolation and removal from blood, in terms of bacterial load by compartment, was assessed in both rodent and human mathematical models.

Analyzing results from this model of multi-organ infection with a PBPK mathematical approach provides an opportunity to evaluate the efficacy of extracorporeal bacterial separation from the bloodstream in combination with various antibiotic treatment regimens. For the first time, a modeling approach based on experimentally obtained data allows quantitative exploration of the impact of extracorporeal bacterial separation in combination with antibiotic treatment.

## Results and Discussion

### Time Course of Bacterial Infection in Immunonormal Rodents

**Fig 1:**
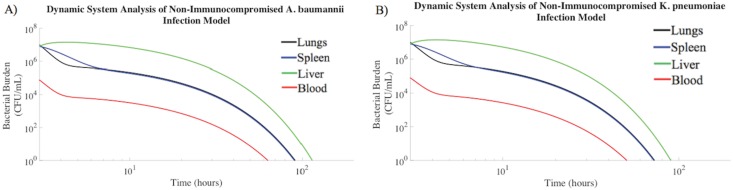
Bacterial burden decreased over time in tissue compartments of healthy rodents. Immunonormal rodents intratracheally inoculated with (A) *A*. *baumannii* (10^7^ CFU/mL) or (B) *K*. *pneumoniae* (10^7^ CFU/mL) were modeled. The median numbers of bacteria in each compartment observed experimentally were used as the initial conditions for these simulations[21,24–27], and trajectories were generated using the parameter estimates described in the Materials and Methods.

Non-immunocompromised rodents challenged intratracheally with a prepared inoculum of either *A*. *baumannii* 10^7^ CFU/mL or *K*. *pneumoniae* 10^7^ CFU/mL[16,17] were modeled. The overall bacterial burden for both Gram-negative species decreased over time in all compartments, although at different rates (Fig 1A and 1B). The blood compartment of the non-immunocompromised rodent mathematical model required the least amount of time to clear bacteria to a negligible amount (≤ 1 CFU/mL), followed by the spleen, lungs, and liver. The bacterial clearance trends shown in Fig 1A and 1B were compared to experimental studies, in which total bacterial burden was reported for the liver, spleen, lungs, and blood of non-immunocompromised rodents following intratracheal bacterial challenge[18–23]. Within 24 hours of intratracheal bacterial challenge, the total bacterial uptake by the liver was the largest, and was followed by the spleen, lungs, and blood[19]. The results of our model were in agreement with experimental results previously described[18–23]. Bruhn et al. experimentally demonstrated that an *A*. *baumannii* bacterial load of 10^4^ CFU/mL within the blood compartment of a normal rodent model was reduced to negligible bacterial densities at a rate of -0.06 log_10_ CFU/mL/h[24]. This *A*. *baumannii* clearance rate, characteristic of the non-immunocompromised rodent blood compartment, was in agreement with in our model predictions (Fig 1A). The blood compartment bacterial load dropped to half maximal concentration within approximately 40 hours of the onset bacterial challenge. This rate of bacterial clearance within the blood compartment was equivalent to -0.06 log_10_ CFU/mL/h. Also, Guo et al. have reported that normal rodents infected intratracheally with an *A*. *baumannii* bolus clear the infection from the lungs at a rate of –0.1 log_10_ CFU/mL/h[20]. The average *A*. *baumannii* clearance rate within the lung compartment of the dynamic model was also -0.1 log_10_ CFU/mL/h (Fig 1A), corresponding to published experimental data. Furthermore, the *A*. *baumannii* bacterial burden within the liver of a normal rodent decreased at a rate of -0.06 log_10_ CFU/mL/h[21], which was the same liver clearance rate produced by the mathematical modeling results.

The mathematical model predictions were in quantitative agreement with experimental studies conducted by other laboratories. The model includes numerous physiological and microbiological parameters, which were chosen from first principles, not mathematical estimations. The agreement of model predictions with experimental data supports the validity of the model structure as representative of bacteremia. The kinetic profiles of *A*. *baumannii* and *K*. *pneumoniae* concentration by tissue were similar for each set of assumptions evaluated in this work. Therefore, the remaining analysis of pathogenesis kinetics is described only for *A*. *baumannii*. The similar *K*. *pneumoniae* data is available in the supplementary data.

Fig 1 suggests that an immunonormal rodent suppresses bacterial growth, with bacterial clearance from each compartment following a pattern of exponential decay. Model initial conditions were 3 hours post-inoculation, focusing the modeling effort on the slower processes of bacterial removal rather than the rapid distribution of bacteria[28]. Bacteria rapidly spread from the lungs to the bloodstream, which resulted in bacterial transport to other organs. The hematogenous spread of bacteria to other organ sites initially caused a rapid decrease in the bacterial burden of the blood compartment, but spread of infection increased the time required to suppress the infection.

### Time Course of Bacterial Infection in Immunocompromised Rodents

**Fig 2:**
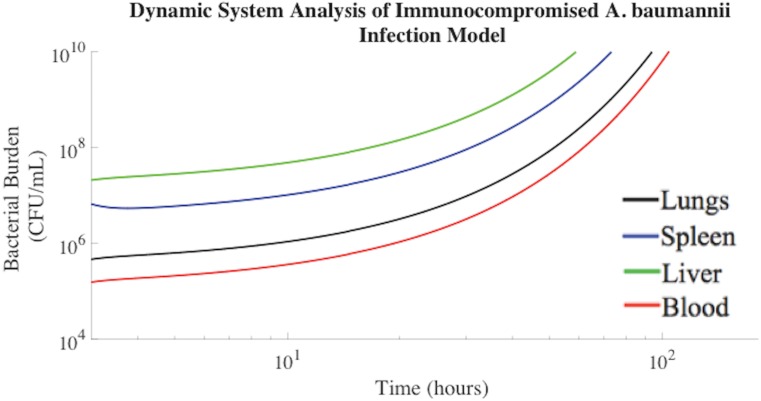
Bacterial time in neutropenic rodents until reaching a lethal *A*. *baumannii* concentration. The median numbers of bacteria in each compartment observed experimentally in previous literature were used as the initial conditions for these simulations[25,35], and trajectories were generated using the parameter estimates shown in Materials and Methods.

*A*. *baumannii* and *K*. *pneumoniae* Gram-negative bacteria frequently cause sepsis in immunocompromised, neutropenic, elderly, and chronically ill individuals[29]. To better evaluate such cases, we modeled the onset of symptomatic bacteremia in neutropenic, immunocompromised rodents. Without treatment, the overall bacterial burden increased in neutropenic rodents until reaching a bacterial load associated with death[30,31]. This experimental condition was modeled in Fig 2. The fitted model agreed with published experimental data regarding bacterial burden over time in an untreated, immunocompromised rodent model[20]. Previously published experimental data demonstrated that a pulmonary *A*. *baumannii* bacterial burden within an immunocompromised rodent increased at a rate of +0.04 log_10_ CFU/mL/h[20,28], which was consistent with the mathematical modeling results (Fig 2). Modeling results also suggest that the *A*. *baumannii* bacterial burden associated with the blood compartment increased at a rate of +0.05 log_10_ CFU/mL/h, which was consistent with the experimental data published by Bruhn et al[24]. Approximately 100 hours following intratracheal exposure to 10^7^ CFU/mL *A*. *baumannii*, neutropenic rodents succumb to the bacterial challenge[32,33]. This was consistent with the lethal bacterial concentration of 10^10^ CFU/mL at 96 hours predicted by mathematical modeling[31–34].

The bacterial burden increases within the lungs, blood, and spleen compartments of Fig 2 are consistent with changes in bacterial transport out of the bloodstream, not simply suppressed bacteria clearance. Predictions obtained for the untreated, immunocompromised rodent model generated bacterial concentrations known to be lethal. Blood flow through the bacterial separation device compartment was set to zero in this scenario. All modeling parameters acquired and implemented were gathered directly from published experimental results and can be found in the Materials and Methods. Solutions from this multi-compartmental model were obtained in the absence of fitting parameters and mimicked experimental data, supporting the validity of the model.

### Efficacy of Antibiotic Administration in Infected, Immunosuppressed Rodents

The most common treatment for symptomatic bacteremia is empiric, broad-spectrum antibiotic therapy. Therefore, colistin methanesulfonate antibiotic administration was incorporated into our mathematical model following the presentation of symptomatic bacteremia, which was presumed to occur when the bacterial concentration in the lungs reached 10^7^ CFU/mL[28] and bacteria were completely distributed throughout all compartments. Bacteria rapidly distribute throughout the blood, liver, lungs, and spleen following exposure[18].

**Fig 3:**
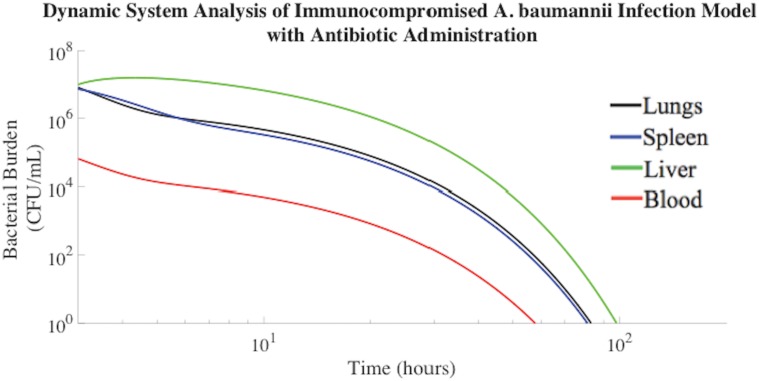
*A*. *baumannii* burden decreased post inoculation in immunocompromised rodents treated with colistin methanosulfate (3 mg/kg). The associated parameters are shown in Materials and Methods.

Following simultaneous intratracheal infusion of 10^7^ CFU/mL *A*. *baumannii* and 3 mg/kg colistin methanosulfate, *A*. *baumannii* was cleared from the blood of the neutropenic rodent mathematical model within 55 hours (Fig 3). The rate constants pertaining to bacterial clearance following colistin methanosulfate administration were based on experimental data gathered experimentally by Pantopoulou et al. and Montero et al[32,36], as described in the Materials and Methods. The mathematical modeling (Fig 3) agreed with experimental data regarding the effects of colistin methanosulfate administration (3 mg/kg) on an *A*. *baumannii* bacterial burden in neutropenic rodent models, clearing the bacterial infection from the blood at a rate of -0.09 log_10_ CFU/mL/h[32,36].

The area under the curve (AUC) was computed for each compartment displayed in Fig 3. The AUC reflects the total, time-dependent bacterial load experienced by each compartment of our model. Of note, the rodent mathematical model suggests that the liver consistently experienced the highest total bacterial burden, approximately an order of magnitude greater than the lungs, as indicated in Table 1. This result is consistent with the severe side effects observed during antibiotic treatment for symptomatic bacteremia, especially in immunocompromised and chemotherapy patients.

**Table 1:** *A*. *baumannii* burden experienced by immunocompromised rodent model administered colistin antibiotic.

Compartment	*A*. *baumannii* ((CFU/mL)*h)
**Lungs (L)**	1.41E7
**Spleen (S)**	1.38E7
**Liver (H)**	1.27E8
**Blood (B)**	1.36E5

### Extracorporeal Bacterial Separation from Blood

Kang et al. have described the development of an extracorporeal bacterial separation device that can rapidly remove bacteria from blood for sepsis treatment[37]. The dialysis-like device employs magnetic nanoparticles functionalized with a bacteria-targeting ligand to magnetically capture pathogens, including multi-drug resistant bacteria, from flowing blood in a microfluidic device. Experimental results using this magnetic nanoparticle-based bacterial separation device suggested that it significantly reduced the levels of bacteria in the bloodstream of a rodent model. It was hypothesized that the spread of bacteria to distal organs would be significantly lowered with the decrease in bacterial concentration in the bloodstream[37]. Also, it was postulated that broad-spectrum antibiotic therapy, such as colistin methanosulfate, could be co-administered with this bacterial separation therapy, resulting in faster bacterial clearance rates.

Magnetic nanoparticle-based extracorporeal bacterial separation could prove to be an effective adjuvant therapy for sepsis treatment. To test this hypothesis, an extracorporeal bacterial separation device was incorporated into the kinetic mathematical model, integrating predictions of magnetic bacteria separation under microfluidic flow in combination with colistin methanosulfate antibiotic treatment. The approach was used to explore the possible benefits of magnetic nanoparticle-based bacterial separation from blood in conjunction with antibiotic treatment on the time-dependent bacterial load among organs during a symptomatic bacteremia episode.

### Bacterial Separation Combined with Antibiotic Therapy

**Fig 4:**
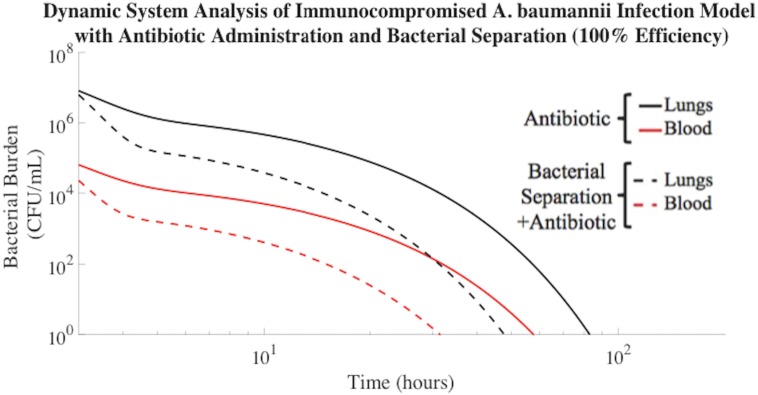
Bacterial separation (100% efficiency) combined with antibiotic administration improved *A*. *baumannii* clearance rate. *A*. *baumannii* cleared from the blood compartment of the immunocompromised rodent model in 28 h when implementing bacterial separation (100% efficiency) and antibiotic treatment. Antibiotic administration alone resulted in bacterial clearance from the blood compartment in 55 h.

The predicted effect of extracorporeal bacterial separation for the purpose of clearing *A*. *baumannii* from the blood is demonstrated in Fig 4. The addition of magnetic nanoparticle-based bacterial separation operating at an ideal 100% efficiency and processing one-fifth of the total blood volume of a rodent per hour, in conjunction with antibiotic treatment, resulted in *A*. *baumannii* clearance from the blood compartment in 27 hours less time than antibiotic treatment alone (Fig 4). Therefore, the bacterial clearance rate associated with this combined treatment was 49% faster than antibiotic treatment alone (Table 2). This is a significant result because for each hour that a septic patient is not effectively treated, the risk of mortality increases by 7.6%[38].

**Table 2:** *A*. *baumannii* clearance (≤1 CFU/mL) time improved upon addition of 100% efficient bacterial separation.

Compartment	Antibiotic Treatment (h)	100% Bacterial Separation Efficiency from Blood + Antibiotic Treatment (h)	% Decrease of Required Treatment Time with Bacterial Separation Implemented [(Antibiotic Treatment—100% Bacterial Separation) /(Antibiotic Treatment)] (%)
**Lungs (L)**	80 h	43 h	46%
**Spleen (S)**	77 h	43 h	44%
**Liver (H)**	95 h	60 h	37%
**Blood (B)**	55 h	28 h	49%

The AUCs of Fig 4 were also evaluated. The model suggested that the liver experienced a 52% reduction in AUC with the addition of bacterial separation in the presence of antibiotic (Table 3). With bacterial separation occurring at 100% efficiency, the extracorporeal bacterial separation device had a significant impact on overall bacterial clearance and diffusion rates. The total bacterial burden in the blood compartment decreased by an order of magnitude compared to antibiotic treatment alone. Also, the incorporation of the 100% effective bacterial separation device into the model inhibited bacterial proliferation and diffusion from the initial inoculation point of the lungs, thereby lowering the overall bacteria burden in all compartments.

**Table 3:** Bacteria separation in immunocompromised *A*. *baumannii* rodent model reduced bacterial burden experienced.

Compartment	Antibiotic Treatment Alone ((CFU/mL)*h)	100% Bacterial Separation Efficiency + Antibiotic ((CFU/mL)*h)	% Decrease of AUC with Bacterial Separation Implemented [(Antibiotic Treatment—100% Bacterial Separation) / (Antibiotic Treatment)] (%)
**Lungs (L)**	1.41E7	3.94E6	72%
**Spleen (S)**	1.38E7	7.33E6	47%
**Liver (H)**	1.27E8	6.11E7	52%
**Blood (B)**	1.36E5	2.03E4	85%

### Device Optimization Based on Bacterial Separation Efficiency

**Fig 5:**
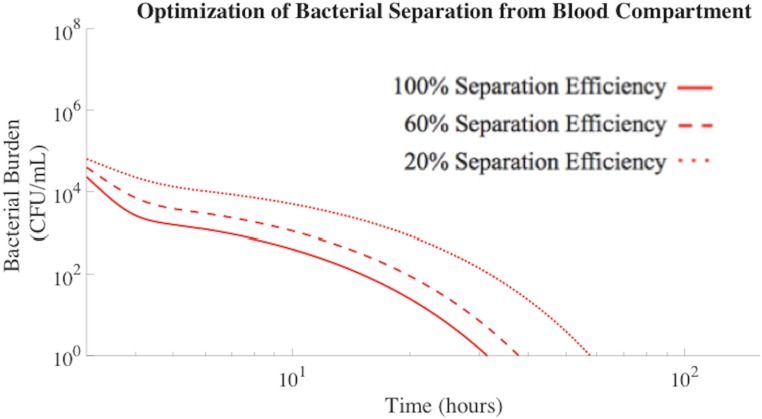
Bacterial separation (100% and 60% efficiency) improved *A*. *baumannii* clearance rates from the blood compartment. 20% bacterial separation efficiency was not efficient enough to impact the overall bacterial clearance rate and resulted in the same clearance rates as antibiotic treatment alone.

Bacterial separation efficiency has a significant impact on the rate of bacteria removal from the blood compartment (Fig 5). As previously displayed in Table 2, 100% separation efficiency promoted the clearance of *A*. *baumannii* from the blood in 27 hours less time than antibiotic treatment alone. To explore the role of separation efficiency on predicted bacterial load *in vivo*, model analysis was carried out for separation efficiencies of 60% and 20%. This approach was intended to inform the design constraints of extracorporeal bacteria separation devices for impact in complex living systems. 60% bacterial separation efficiency resulted in clearance of *A*. *baumannii* from the blood compartment in 20 hours less time that antibiotic treatment alone (Table 4). As the separation efficiency further decreased to 20%, bacterial removal became ineffective. Therefore, device design features that impact bacterial separation efficiency and nonlinearly influence clearance must be considered for proposed applications.

**Table 4:** *A*. *baumannii* clearance (≤1 CFU/mL) time in immunocompromised rodent model accelerated with improved bacterial separation efficiencies.

Compartment	100% Separation Efficiency (25 nm) + Antibiotic (h)	60% Separation Efficiency (30 nm) + Antibiotic (h)	20% Separation Efficiency (43 nm) + Antibiotic (h)	Antibiotic Treatment (h)
**Lungs (L)**	43 h	51 h	80 h	80 h
**Spleen (S)**	43 h	52 h	78 h	78 h
**Liver (H)**	60 h	67 h	95 h	95 h
**Blood (B)**	28 h	35 h	55 h	55 h

Total bacteria separation efficiencies produced by the mathematically modeled extracorporeal separation devices were dependent on nanoparticle size. As the nanoparticle radius increased, the bacterial separation efficiency decreased, resulting in slower clearance of bacteria from blood (Fig 5). The collision rate constant decreased as the nanoparticle radius increased, resulting in less total nanoparticle interaction with, and binding to, bacteria[13]. Once the nanoparticle radius exceeded approximately 40 nm, the efficiency of bacterial binding and capture was not significant enough to impact the overall rate of bacterial clearance. The optimal nanoparticle size for bacteria capture and removal was estimated to be 25 nm or less, which directly corresponded to previously published literature[13]. The reduced mass of smaller nanoparticles, however, decreases the magnetic attraction force[13]. Thus, careful attention to the design of the separation device is required to achieve efficiency compatible with predicted improvement in overall bacterial clearance. Therefore, multiple, interconnected nanotechnology design features are important considerations in magnetic nanoparticle-based bacterial separation devices.

The impact of bacterial separation efficiency was evaluated by the mathematical model and reported in terms of resultant total bacterial load, or AUC, experienced by each tissue compartment of an *A*. *baumannii* infected immunocompromised rodent. As the nanoparticle radius increased above 25 nm, the overall separation efficiency characteristic of the extracorporeal bacterial separation device decreased. Table 5 demonstrated that as the nanoparticle radius increased above 25 nm, the total bacterial burden experienced by each compartment also increased. All other parameters held constant, a nanoparticle radius of 43 nm is equivalent to a 20% separation efficiency, which has no significant effect on the AUC compared to antibiotic treatment alone. Therefore, in order for magnetic nanoparticle-based bacterial separation devices to aid in the reduction of the total bacterial burden within each compartment, the efficiency of bacterial separation from the blood compartment must exceed 20%.

**Table 5:** Bacteria separation efficiencies greater than 20% reduced bacterial burden in *A*. *baumannii* immunocompromised rodent model.

Compartment	100% Separation Efficiency (25 nm) + Antibiotic ((CFU/mL)*h)	60% Separation Efficiency (30 nm) + Antibiotic ((CFU/mL)*h)	20% Separation Efficiency (43 nm) + Antibiotic ((CFU/mL)*h)	Antibiotic Treatment Alone ((CFU/mL)*h)
**Lungs (L)**	3.94E6	6.10E6	1.41E7	1.41E7
**Spleen (S)**	7.33E6	8.70E6	1.38E7	1.38E7
**Liver (H)**	6.11E7	7.37E7	1.20E8	1.27E8
**Blood (B)**	2.03E4	4.48E4	1.36E5	1.36E5

### Bacterial Separation for Treatment of Symptomatic Bacteremia in Human Model

The use of extracorporeal bacterial separation for treatment of symptomatic bacteremia in humans has never been explored experimentally or by mathematical modeling. The successful rodent model was modified to explore the possible benefits of magnetic nanoparticle-based extracorporeal bacterial separation in humans during an episode of symptomatic bacteremia. By incorporating human parameters (Materials and Methods) into the model and adjusting for the tissue volume differences of humans compared to rodents, we were able to estimate the impact of bacterial separation, in conjunction with antibiotic treatment. The model extrapolation to humans has known, and, likely, unknown limitations. However, this approach is a first step towards an understanding of the extracorporeal bacterial clearance necessary for biological impact.

**Fig 6:**
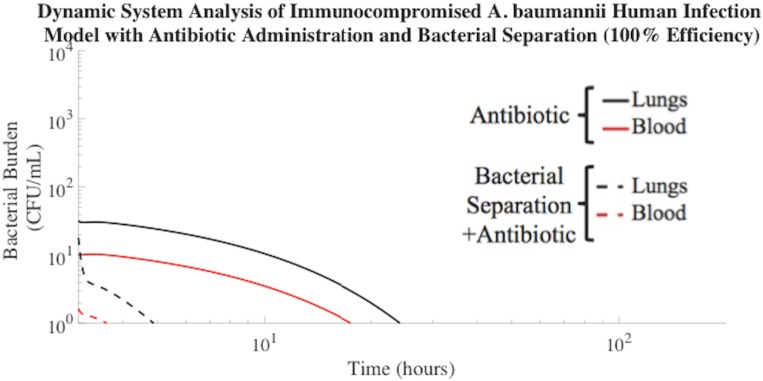
Bacterial separation (100% efficiency) with antibiotic treatment improved *A*. *baumannii* clearance from human blood compartment. This combination treatment resulted in *A*. *baumannii* clearance (≤1 CFU/mL) from the blood compartment in 1 h. The time required for *A*. *baumannii* to be cleared to a negligible concentration (≤ 1 CFU/mL) from the blood compartment of the human model with antibiotic administration alone was 15 h.

Symptomatic bacteremia occurs in an adult humans when the bacterial burden reaches approximately 10 CFU/mL in the bloodstream or 10^3^ CFU/mL in the lungs[39–41]. Therefore, our model incorporated these parameters as markers to indicate the time at which to apply extracorporeal bacterial separation and antibiotic treatment. Results suggested that *A*. *baumannii* clearance from the blood compartment was reduced by 14 hours with the addition of 100% efficient extracorporeal bacterial separation treatment, compared to antibiotic treatment alone (Fig 6). The flow rate through the bacterial separation compartment was programmed to process one-fifth of the total human blood volume per hour, similar to the blood flow rates used during kidney dialysis in humans. Based on the AUCs of Fig 6, the combination therapy approach decreased the total bacterial burden in the model compartments, with the most significant difference in the blood compartment (Table 6).

**Table 6:** Bacteria separation (100% efficiency) in *A*. *baumannii* human model reduced bacterial burden experienced.

Compartment	100% Bacterial Separation Efficiency + Antibiotic ((CFU/mL)*h)	Antibiotic Treatment Alone ((CFU/mL)*h)
**Lungs (L)**	5.30E1	2.67E2
**Spleen (S)**	2.66E2	2.25E3
**Liver (H)**	1.81E3	1.28E4
**Blood (B)**	0.326E1	7.53E1

This work is the first to estimate, by mathematically models, bacterial load as a surrogate of the ultimate biological impact of infection. Bacterial load, or AUC, is directly correlated to the progression of symptomatic bacteremia and death[42]. Therefore, extracorporeal bacterial separation devices designed to operate at high efficiencies would not only significantly reduce the total bacterial load, but may also inhibit the progression of symptomatic bacteremia. Inhibition of disease progression would provide additional time to identify ideal therapies for infected patients, while reducing the mortality rates associated with symptomatic bacteremia.

### Bacterial Separation for Treatment of Multi-Drug Resistant Bacteremia in Human Model

Bacterial species identification in the clinical setting takes one to three days, a time during which the patient is treated with broad-spectrum antibiotics. This method is not optimal, especially for patients suffering from MDR infections. Symptomatic bacteremia caused by MDR bacteria is one of the most critical public health issues, even in first-world countries[43]. The increasing frequency of MDR bacterial strains is leading into the ‘post-antibiotic era’.

**Fig 7:**
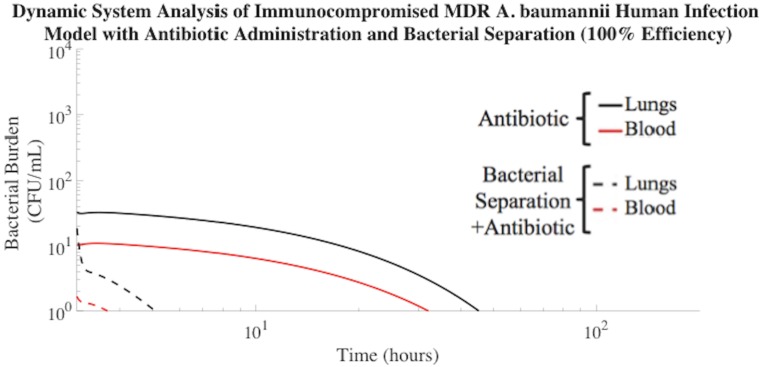
Treatment of MDR *A*. *baumannii* human model using 100% efficient bacterial separation with antibiotic treatment. Using this combination therapy, MDR *A*. *baumannii* clearance (≤ 1 CFU/mL) from the blood compartment of the human mathematical model occurred in 1 h. The time required for MDR *A*. *baumannii* to be cleared to a negligible concentration (≤1 CFU/mL) from the blood compartment with antibiotic administration alone was 29 h.

MDR-associated symptomatic bacteremia was mathematically modeled by assuming that the MDR *A*. *baumannii* were 50% less susceptible to antibiotic treatment than non-MDR *A*. *baumannii*. Bacterial infection that is 0% susceptible to antibiotic treatment behaves similarly to the trajectory shown in Fig 2 of untreated infection, whereas infection that is 100% susceptible to antibiotic treatment was shown in Fig 3. Choosing a MDR *A*. *baumannii* susceptibility of 50% allows for quantitative evaluation of model sensitivity to the degree of bacterial antibiotic resistance. Fig 7 described the clearance of MDR *A*. *baumannii* using the additional assistance of 100% efficient extracorporeal bacterial separation, compared to antibiotic treatment alone. The addition of extracorporeal bacterial separation operating at 100% efficiency resulted in MDR *A*. *baumannii* clearance from the blood of a human mathematical model in 1 h, which was 97% faster than antibiotic treatment alone. Antibiotics prove less effective in the treatment of MDR symptomatic bacteremia, which highlights the need for alternative treatment methods. Bacterial separation treatment methods have been shown to bind and remove multiple clinical isolates of antibiotic-resistant organisms[37]. This supports the development of new engineering-based bacterial separation devices to combat MDR bacteria and delay progression to septic shock.

Our mathematical modeling work established that extracorporeal bacterial separation reduced the total bacterial burden in the bloodstream of a bacteremic subject by an order of magnitude, compared to antibiotic treatment alone. This correlated to a 49% reduction in non-MDR bacterial exposure time due to the addition of extracorporeal bacterial separation therapy. Efficient bacterial separation also reduced the spread of bacteria to distal sites. Results further indicated that engineering-based bacterial separation devices could offer a particularly effective therapeutic strategy for patients suffering from MDR infections that render existing drug therapies inadequate. The addition of extracorporeal bacterial separation resulted in MDR bacterial clearance from the blood of a human mathematical model in 97% less time than antibiotic treatment alone. This was a notably important prediction because decreasing the length of bacterial infection significantly reduces the risk of mortality associated with symptomatic bacteremia[38]. The lack of antibiotics in the developmental pipeline, combined with the increasing rate of MDR, has created a dire need for the discovery of new therapies effective against MDR bacterial infections. Therefore, development of mathematical models is necessary to rapidly determine the effectiveness of new treatment therapies, such as extracorporeal bacterial separation. Our work was the first dynamic mathematical model to demonstrate the potential usefulness of extracorporeal bacterial separation for the treatment of symptomatic bacteremia and its MDR counterpart.

Engineers have had a profound impact on the development of disease treatment systems, such as kidney dialysis devices. It is clear that engineers also have a role to play in the design of extracorporeal bacterial separation devices. To aid in the optimization of bacterial separation device design, more experimental data quantifying bacterial load in living systems must be gathered to better understand the correlation between bacterial load and patient outcome.

Currently, data describing bacterial load in rodent systems is sparse, and corresponding human data is even less prevalent. These are key pieces of information that must be obtained in order to confidently design new treatment methods that reduce total bacterial burden, duration of infection, and the ‘post-antibiotic era’ threat. Designing extracorporeal bacterial separation devices that remove bacteria from the blood at a rate deemed biologically relevant by this information may inhibit the progression of symptomatic bacteremia, decrease overall mortality rates, and help combat MDR bacterial infections.

## Materials and Methods

### Kinetic Model and Parameter Values

**Fig 8:**
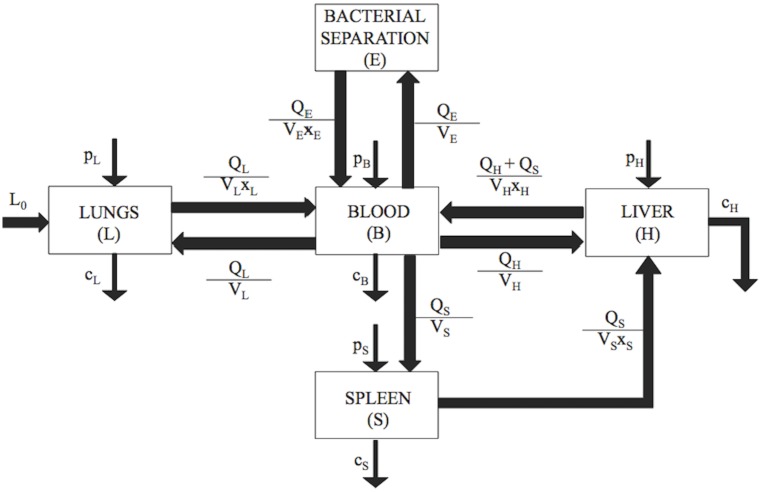
The five-compartment kinetic model describing bacterial pathogenesis. Intratracheal instillation of a Gram-negative bacteria bolus initially occurred in the lung compartment, with initial concentration *L*_*0*_ (CFU mL^-1^). Bacterial proliferation rates (*p*, h^−1^), clearance rates (*c*, h^−1^), and transport rates between compartments were included in the model schematic. The rate of bacterial transport between compartments was represented as a function of blood flow rate per compartment volume (*Q*/*V*, mL h^-1^), modified by an experimentally determined partitioning coefficient (*x*, dimensionless).

A five-compartment kinetic model was developed to explore the pathogenesis kinetics and treatment of symptomatic bacteremia (Fig 8). It was assumed that infection occurred via intratracheal instillation of a 10^7^ CFU/mL Gram-negative bacteria (A*cinetobacter baumannii* or *Klebsiella pneumoniae*) bolus into the lung compartment. This is a common bacterial concentration used to establish and study rodent bacteremia models[16]. Also, the most common site of infection leading to bacteremia in humans is the lungs[44,45]. Bacterial exchange among all five compartments, which included the lungs, spleen, liver, blood, and bacterial separation device, was programmed as specified by the model construction and parameters extracted from experimental studies of bacteremia. The lungs, liver, spleen, and blood were included because all have experimentally demonstrated the most significant and rapid uptake of blood-borne bacteria[18]. Kinetics were assumed to be first order. Bacteria proliferated (*p* (h^-1^)) within the lungs, spleen, liver, and blood, and were cleared (*c*) by immunological interactions at rates (h^-1^) specific to each compartment. No experimental evidence was available to distinguish *p* from *c in vivo*, therefore, the two terms were considered as a single net bacterial growth rate (*G*_*x*_ (h^-1^)). Bacterial transport between compartments was represented as a function of the blood flow rate, *Q*, the compartment volume, *V*, and a partitioning coefficient, *x*[18]. The partition coefficient represented the ratio of bacteria concentration between compartments and was proportional to the concentration of bacteria in the donor compartment[18]. Bacterial separation efficiency, *f(r*_*f*_*)*, represented the percent of target bacterial cells separated by the bacterial separation compartment (CFU mL^-1^) per total number of target bacterial cells (CFU mL^-1^).

Five first-order homogenous ordinary differential equations (ODEs) were used as a model representation of the physiological features of the system. The system of equations was based on previously published pharmacokinetic models[18,46]. Each autonomous ODE represented the instantaneous rate of change of bacteria concentration in the respective compartment,

$$\frac{dL}{dt}=\left(\left(G_{L}\right)*L\right)+\left(\frac{Q_{L}}{V_{L}}*B\right)-\;\left(\frac{Q_{L}}{V_{L}x_{L}}*L\right)$$ (1)

$$\frac{dS}{dt}=\left(\left(G_{s}\right)*S\right)+\left(\frac{Q_{s}}{V_{s}}*B\right)-\;\left(\frac{Q_{s}}{V_{s}x_{s}}*S\right)$$ (2)

$$\frac{dH}{dt}=\left(\left(G_{H}\right)*H\right)+\left(\frac{Q_{H}}{V_{H}}*B\right)+\left(\frac{Q_{s}}{V_{s}x_{s}}*S\right)-\left(\frac{Q_{H}+Q_{s}}{V_{H}x_{H}}*H\right)$$ (3)

$$\frac{dE}{dt}=\left(\frac{Q_{E}}{V_{E}}*B*f\left(r_{f}\right)\right)-\;\left(\frac{Q_{E}}{V_{E}x_{E}}*E*\left(1-f\left(r_{f}\right)\right)\right)$$ (4)

$$\frac{dB}{dt}=\left(\left(G_{B}\right)*B\right)+\left(\frac{Q_{H}+Q_{s}}{V_{H}x_{H}}*H\right)+\left(\frac{Q_{L}}{V_{L}x_{L}}*L\right)+\;\left(\frac{Q_{E}}{V_{E}x_{E}}*E*\left(1-f\left(r_{f}\right)\right)\right)-\left[\left(\frac{Q_{H}}{V_{H}}+\;\frac{Q_{s}}{V_{s}}+\;\frac{Q_{L}}{V_{L}}+\left(\frac{Q_{E}}{V_{E}}*f\left(r_{f}\right)\right)\right)*B\right]$$ (5)

where *L*, *S*, *H*, *E*, and *B* were the bacterial concentrations (CFU mL^-1^), in the lungs, spleen, liver, extracorporeal bacterial separation device, and blood, respectively. The model was designed such that bacteria were transported from the spleen into the liver via hepatic portal circulation. Liver (H, hepatic) circulation was divided into splenic portal venous blood and the combination of hepatic arterial flow and its tributaries. Mouse and human organ mass values were based on published literature and were used to describe organ volumes, *V*[18]. Organ blood flow rates, *Q*, and blood:organ partition coefficients, *x*, were also gathered from previously published data[18]. Empirical values used to describe compartmental volumes, blood flow rates, and partition coefficients can be found in Tables 7–9.

**Table 7:** Model Parameters, Rodent[18].

Compartment	Blood Flow Rate, *Q* (mL min^-1^ g^-1^)	Volume, *V* (g or mL)
**Lungs (L)**	6.84	2.10
**Spleen (S)**	1.20	0.15
**Liver (H)**	1.50	1.99
**Extracorporeal Bacterial Separation Device (E)**	Variable	0.032

**Table 8:** Model Parameters, Human.

Compartment	Blood Flow Rate, *Q* (mL min^-1^ g^-1^)	Volume, *V* (g or mL)
**Lungs (L)**	1.14[47]	1315[48]
**Spleen (S)**	1.97[49]	127[48]
**Liver (H)**	0.97[50]	830[48]
**Extracorporeal Bacterial Separation Device (E)**	Variable	90

**Table 9:** Partition Coefficient Model Parameters[18].

Compartment	Partition Coefficient, *x* (Immunocompromised)	Partition Coefficient, *x* (Non-Immunocompromised)
**Lungs (L)**	3	93
**Spleen (S)**	28	59
**Liver (H)**	79	749
**Extracorporeal Bacterial Separation Device (E)**	1	1

The rodent model was based on 58.5 ml of blood per kg of bodyweight[51]. A mouse weighing 31 g would have a total blood volume of approximately 1.80 mL. Therefore, subsequent calculations were scaled to account for the 5000 mL average total blood volume of humans.

The net bacterial growth rates, *G*_*x*_, in each compartment of non-immunocompromised subjects, along with immunocompromised neutropenic subjects, can be found in Tables 10 and 11. These values were derived from published literature in which the total bacterial burden was measured in each compartment of a rodent at specified time points. The bacterial burden rates of change, or net bacterial growth rates, were calculated for each compartment using the cited published literature. The calculated rates of change were then implemented as the net bacterial growth rates in the model. The rates of change were estimated by linearization of the experimental data from which rate of change can be calculated, which is equivalent to the slope of the linear approximation. An example of this calculation is shown in the S5 Fig. Similar, time dependent and organ specific bacterial levels have not been reported for human subjects. Therefore, net bacterial growth rates in human compartments were extrapolated from rodent models based on relative compartment characteristics.

**Table 10:** Net bacterial growth rates, *A*. *baumannii*.

Compartment	Bacterial Growth Rate, *G*_*x*_ (h^-1^) (Immunocompromised)	Bacterial Growth Rate, *G*_*x*_ (h^-1^) (Non-Immunocompromised)
**Lung (L), (*G***_***L***_**)**	0.21[20]	-1.74[20,25]
**Spleen (S), (*G***_***S***_**)**	0.14[18]	-0.14[18]
**Liver (H), (*G***_***H***_**)**	0.10[25]	-0.10[21]
**Blood (B), (*G***_***B***_**)**	0.08[24]	-0.17[24]

**Table 11:** Net bacterial growth rates, *K*. *pneumoniae*.

Compartment	Bacterial Growth Rate, *G*_*x*_ (h^-1^) (Immunocompromised)	Bacterial Growth Rate, *G*_*x*_ (h^-1^) (Non-Immunocompromised)
**Lung (L), (*G***_***L***_**)**	0.10[35]	-1.50[26]
**Spleen (S), (*G***_***S***_**)**	0.11[35]	-0.11[27]
**Liver (H), (*G***_***H***_**)**	0.13[35]	-0.15[27]
**Blood (B), (*G***_***B***_**)**	0.15[35]	-0.10[27]

Net bacterial growth rates corresponding to each compartment following colistin antibiotic treatment are displayed in Tables 12 and 13[32,52]. Experimental evidence was not available to distinguish *p* from *c in vivo*. However, it was possible to deduce single net bacterial growth rates (*G*_*x*_ (h^-1^)) by analyzing literature that reported the total bacterial burden in each compartment before and after colistin treatment.

**Table 12:** Net bacterial growth rates, *A*. *baumannii* with colistin[32].

Compartment	Bacterial Growth Rate, *G*_*x*_ (h^-1^) (Immunocompromised + Antibiotic)
**Lung (L), (*G***_***L***_**)**	-0.24
**Spleen (S), (*G***_***S***_**)**	-0.07
**Liver (H), (*G***_***H***_**)**	-0.18
**Blood (B), (*G***_***B***_**)**	-0.05

**Table 13:** Net bacterial growth rates, *K*. *pneumoniae* with colistin[32,52].

Compartment	Bacterial Growth Rate, *G*_*x*_ (h^-1^) (Immunocompromised + Antibiotic)
**Lung (L), (*G***_***L***_**)**	-0.35
**Spleen (S), (*G***_***S***_**)**	-0.10
**Liver (H), (*G***_***H***_**)**	-0.15
**Blood (B), (*G***_***B***_**)**	0.02

Sensitivity analysis based on parameter variation from experiments demonstrates modest changes in the bacterial concentration among compartments as a function of time, but does not influence the conclusions of this study.

### Magnetic Separation Component Design

The bacterial separation (E) component of the model embodied a fluidic device in which continuous magnetic separation of Gram-negative bacteria from non-Newtonian particulate blood flow occurred. The magnetic separation device consisted of two steps, together comprising the total magnetic separation efficiency, *f(r*_*f*_*)*: 1) Gram-negative bacteria–targeted magnetic nanoparticles bound to the bacterial cells and 2) the magnetic separation of nanoparticle-bacteria complexes from the blood. Total magnetic separation efficiency, *f(r*_*f*_*)*, of bacteria from the blood was dependent upon magnetic nanoparticle size, blood viscosity, and magnetic forces. The modeling parameters and equations used to calculate the value of *f(r*_*f*_*)* were based on previous studies[13,53].

Briefly, the value of *f(r*_*f*_*)* was calculated for various nanoparticle sizes by determining the binding kinetics between the magnetic nanoparticles and bacteria. Assuming that all bacteria bound to magnetic nanoparticles were removed by the magnetic fluidic device, the binding efficiency, *x*(*r*f), was represented by

$$x\left(r_{f}\right)=\left(1-\;e^{\left(-c_{e}\left(k_{d}+k_{shear}\right)*b*t\right)}\right)*100$$ (6)

where *b* was the concentration of magnetic nanoparticles, *k*_*d*_ the diffusion collision rate constant, *k*_*shear*_ the shear collision rate constant, and *c*_*e*_ (3.7 * 10^−4^ <dimensionless>) an empirical constant representing the binding efficiency of bacteria to the magnetic nanoparticles in whole blood[13].

Then, the effects of the magnetophoretic separation of the magnetic nanoparticle-bound bacteria from the blood under continuous flow were determined. The magnetic forces acting on the magnetic nanoparticle-bound bacteria directly impacted the magnetic separation efficiency. Therefore, the magnetic separation efficiency was estimated by calculating the magnetic force induced by our defined theoretical parameters,

$$F_{mag}=N*\;\frac{4\pi r_{f}^{3}}{3}*\frac{\Delta \chi }{2\mu_{o}}*B^{2}$$ (7)

where *r*_*f*_ was the radius of a magnetic nanoparticle, *μ*_*o*_ was the magnetic permeability of vacuum (4π * 10^−7^ < *T m A*^*-1*^>), *B*^*2*^ was the magnetic field intensity *(B*^*2*^ = 40 < *T*^*2*^ *m*^*-1*^>), *N* was the number of magnetic nanoparticles bound to the bacteria cell, and Δχ (<dimensionless>) was the volumetric susceptibility of the magnetic nanoparticles[53]. The following conditions were used:

$$\Delta \chi =\;\frac{4*3.5*\;\pi *\;r_{f}^{3}}{3}$$ (8)

$$N=4*\;\rho *\;{\left(\frac{r_{c}}{r_{f}}\right)}^{2}$$ (9)

where *r*_*c*_ was the effective spherical radius of a pathogen (0.5 μm, *A*. *baumannii*[54]; 0.65 μm, *K*. *pneumoniae*[55]) and it was assumed, based on previous literature, that half of the cell surface was covered by magnetic particles (ρ = 0.5 <dimensionless>)[13,53].

Assuming a quasi-static motion,

$$F_{mag}=F_{drag}=6\pi r_{n}\eta v_{mag}$$ (10)

where *r*_*n*_ was the effective hydraulic radius of the bacteria–nanoparticle complex and η was the blood viscosity (4 * 10^−3^ <N s m^-2^>)[13]. The variable *r*_*n*_ can be represented as

$$r_{n}=\;\sqrt[{3}]{r_{c}^{3}+N*r_{f}^{3}}$$ (11)

For small particles, *F*_*mag*_ *= F*_*drag*_ and, therefore, the magnetophoretic velocity (*v*_*mag*_) of the magnetic nanoparticle-bacteria complex can be defined as

$$v_{mag}=\;\frac{r_{n}^{2}N\Delta \chi B^{2}}{9\eta \mu_{o}}$$ (12)

Blood flowing through the magnetic extraction fluidic component at a flow rate of *Q* (L h^-1^) resulted in an average linear velocity (*v*_*1*_) of bacteria labeled with magnetic nanoparticles of

$$v_{1}=\;\frac{Q}{a}$$ (13)

where *a* was the cross-sectional area of the device channel (0.002 m x 0.0006 m; width x height)[13]. The characteristic residence time, *t*_*res*_, of the nanoparticle-bound bacteria in the magnetic fluidic device channel was approximated by

$$t_{res}=\;\frac{L_{L}}{v_{1}}$$ (14)

where *L*_*L*_ was the hypothetical length of the channel (*L*_*L*_ = 0.027 m)[13]. The magnetophoretic transverse time (*t*_*mag*_), the time it takes the nanoparticle-bound bacteria to be extracted from the blood by the magnets of the fluidic device, was described by

$$t_{mag}=\;\frac{L_{h}}{v_{mag}}$$ (15)

where *L*_*h*_ was the height of the magnetic fluidic device channel (0.0006 m)[13].

The magnetic separation efficiency, *m(r*_*f*_*)*, was then estimated by comparing the characteristic residence time (*t*_*res*_) to the time required for the nanoparticle-bound bacteria to be extracted from the blood by the magnets of the fluidic device (*t*_*mag*_)[13].

$$m(r_{f})=100*\;\frac{t_{res}}{t_{mag}}\;,\;t_{res}<\;t_{mag}$$ (16)

$$m(r_{f})=100\;,\;t_{res}\geq \;t_{mag}$$ (17)

Finally, the total magnetic separation efficiency, *f(r*_*f*_*)*, for the two-step process was calculated using

$$f\left(r_{f}\right)=\;x(r_{f})*m(r_{f})$$ (18)

**Fig 9:**
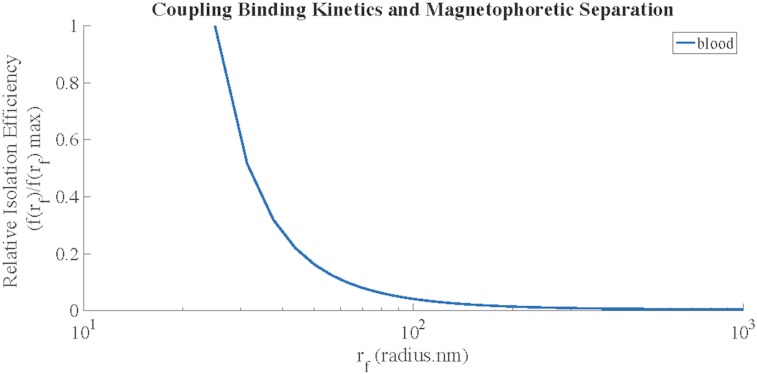
Magnetic separation efficiency, *f(r*_*f*_*)*, of Gram-negative bacteria incubated with bacteria-targeting magnetic nanoparticles in whole blood. In the model predictions, the maximum magnetic separation efficiency occurred using magnetic nanoparticles with a radius of 25nm. The theoretical model prediction was verified by comparison to results published by Kang et al.[13].

Maximum magnetic separation of bacteria from blood was predicted to occur when using magnetic nanoparticles with 25 nm radii. As the magnetic nanoparticle radius increased, the extraction efficiency decreased (Fig 9). Experimental and theoretical results from Kang et al. were used to validate this trend[13].

### Model Analysis

All analysis was written in Matlab R2015b (The Mathworks, Inc, Natick, Mass). The model equations were solved using the appropriate ODE solver in Matlab. The parameters values were verified by fitting the solution of the differential equations to experimental data found in literature.

## Supporting Information

S1 Fig Bacterial burden increased over time in neutropenic rodents until reaching a lethal *K*. *pneumoniae* concentration. The median numbers of bacteria in each compartment observed experimentally in previous literature were used as the initial conditions for these simulations(25,35), and trajectories were generated using the parameter estimates shown in Table 11.
(TIFF)

S2 Fig *K*. *pneumoniae* bacterial burden trajectories post inoculation in immunocompromised rodents treated with colistin sulfate (3 mg/kg). (TIFF)

S3 Fig *K*. *pneumoniae* burden decreased more rapidly with bacterial separation treatment in combination with antibiotic. 100% bacterial separation efficiency, combined with antibiotic administration (colistin methanosulfate, 3mg/kg), resulted in bacterial clearance from the blood compartment in 32 h. This was 29 h faster than antibiotic treatment alone.
(TIFF)

S4 Fig 100% and 60% bacterial separation efficiencies had a significant impact on overall bacterial clearance rates from the blood compartment of immunocompromised *K*. *pneumoniae* rodent model. 20% bacterial separation efficiency was not efficient enough to significantly impact the overall bacterial clearance rate and resulted in the same clearance rates as antibiotic treatment alone.
(TIFF)

S5 Fig The net bacterial growth rates were estimated by linearization of experimental data. The net bacterial growth rate of *A*. *baumannii* in the liver of a non-immunocompromised rodent model is provided as an example calculation [21]. The linearization equation used to calculate all net bacterial growth rates is detailed in S5 Fig.
(TIF)

S1 Table Bacterial burden experienced by immunocompromised *K*. *pneumoniae* rodent model administered antibiotic. (TIFF)

S2 Table 100% efficient bacterial separation accelerates *K*. *pneumoniae* clearance (≤1 CFU/mL) in immunocompromised rodent model. (TIFF)

S3 Table Bacteria separation in immunocompromised *K*. *pneumoniae* rodent model reduced bacterial burden experienced. (TIFF)

S4 Table *K*. *pneumoniae* clearance (≤1 CFU/mL) in immunocompromised rodent model accelerated with improved bacterial separation efficiencies. (TIFF)

S5 Table 60% or greater bacteria separation efficiencies reduced bacterial burden in *K*. *pneumoniae* immunocompromised rodent model. (TIFF)

S1 Text Source Code. (DOCX)
