# Supplementary figures and images for: Dynamic Computational Model of Symptomatic Bacteremia to Inform Bacterial Separation Treatment Requirements
Source: PLoS One. 2016 Sep 22;11(9):e0163167. doi: 10.1371/journal.pone.0163167 (PMC5033423; doi:10.1371/journal.pone.0163167)

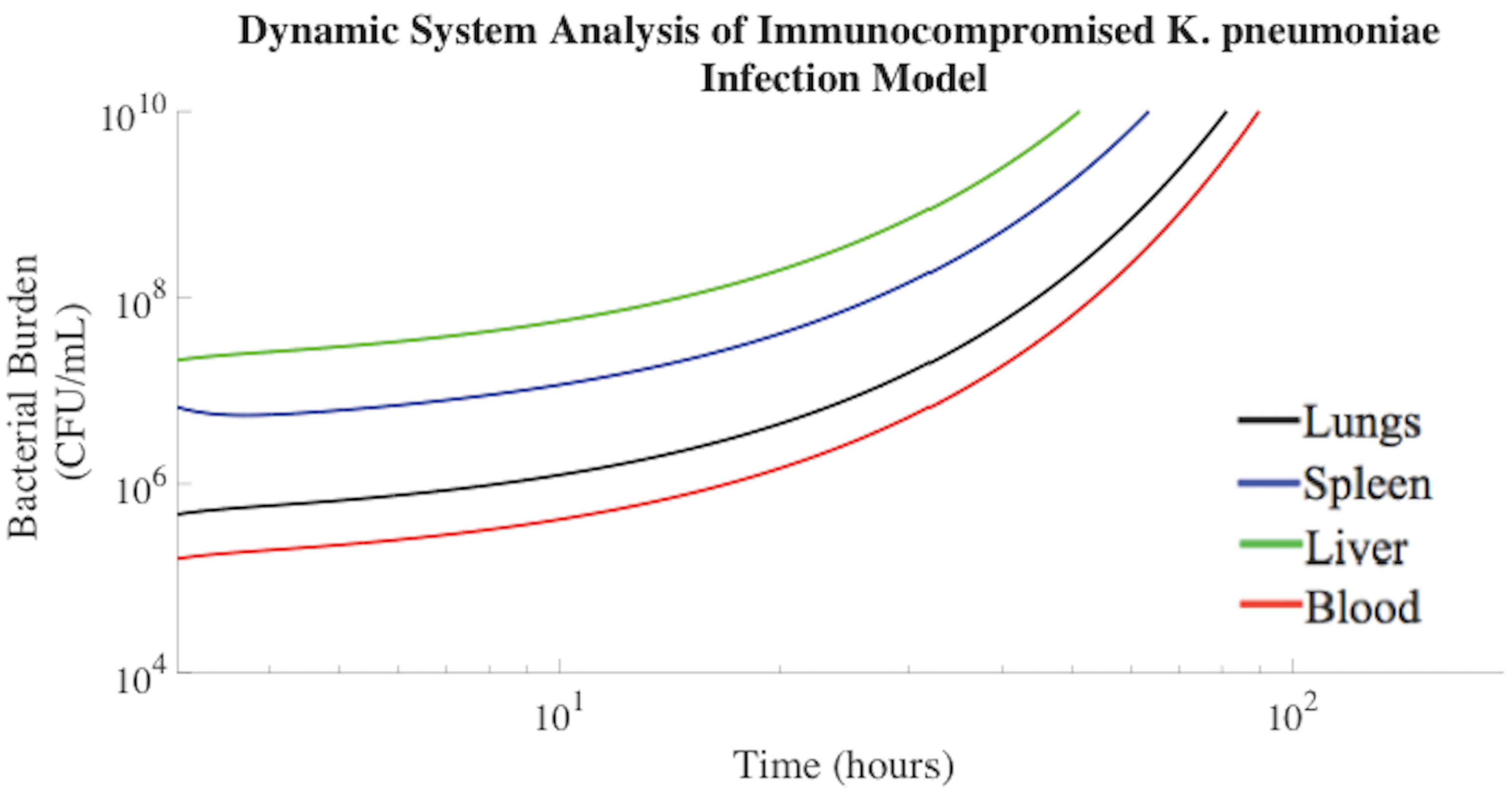

Supplement: S1 Fig — The median numbers of bacteria in each compartment observed experimentally in previous literature were used as the initial conditions for these simulations(25,35), and trajectories were generated using the parameter estimates shown in Table 11. (TIFF) [file pone.0163167.s001.tiff]

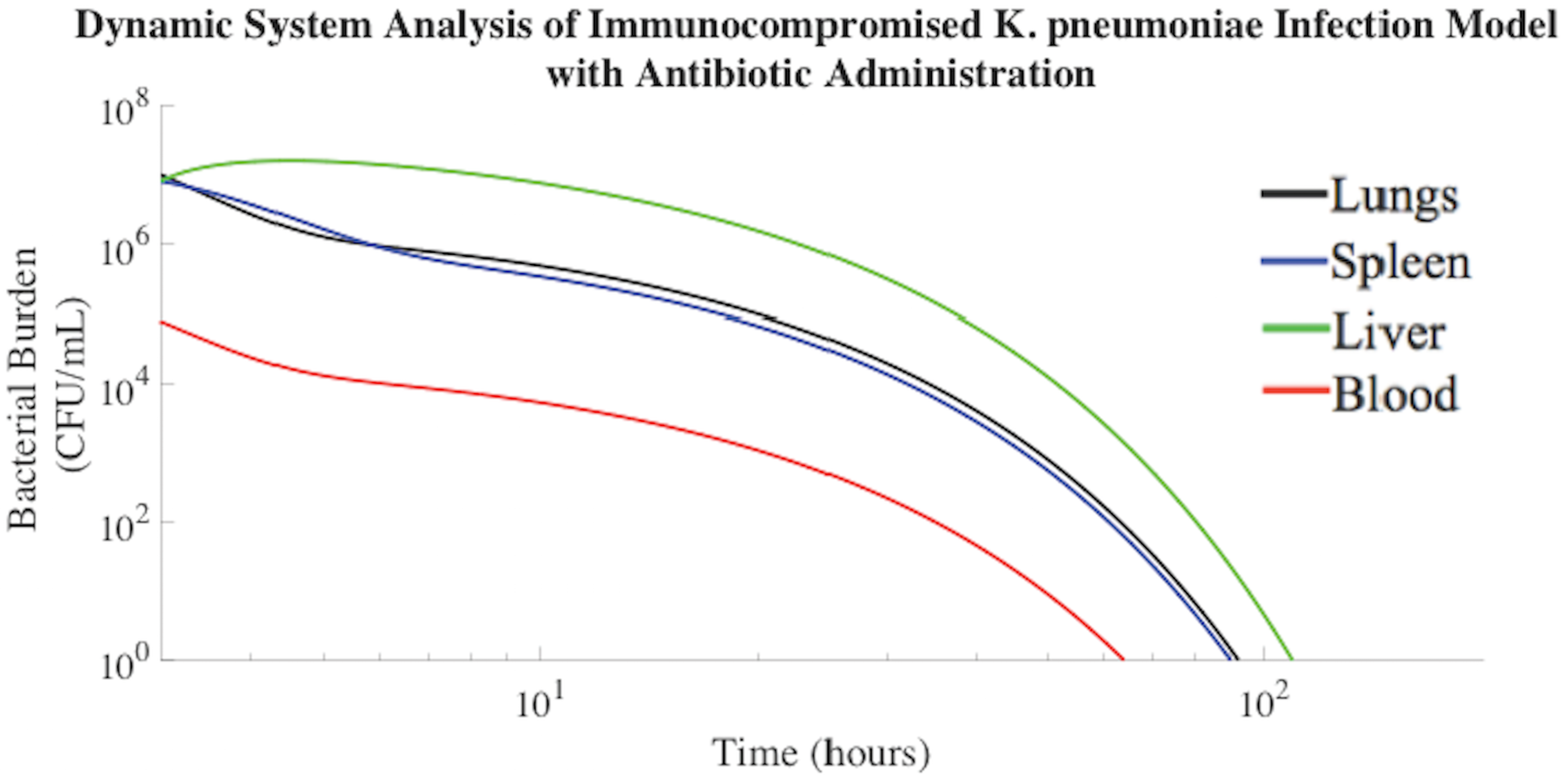

Supplement: S2 Fig — (TIFF) [file pone.0163167.s002.tiff]

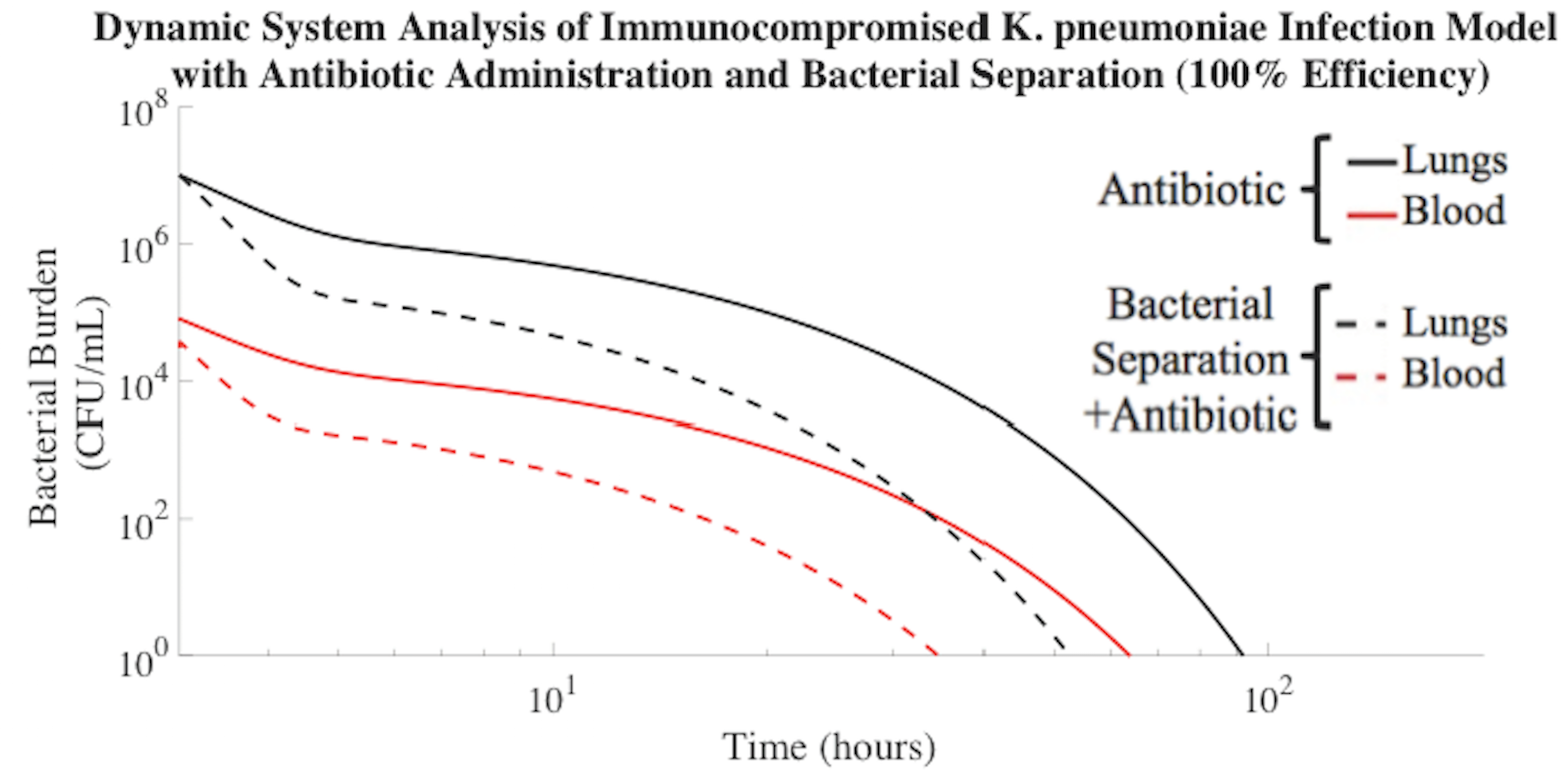

Supplement: S3 Fig — 100% bacterial separation efficiency, combined with antibiotic administration (colistin methanosulfate, 3mg/kg), resulted in bacterial clearance from the blood compartment in 32 h. This was 29 h faster than antibiotic treatment alone. (TIFF) [file pone.0163167.s003.tiff]

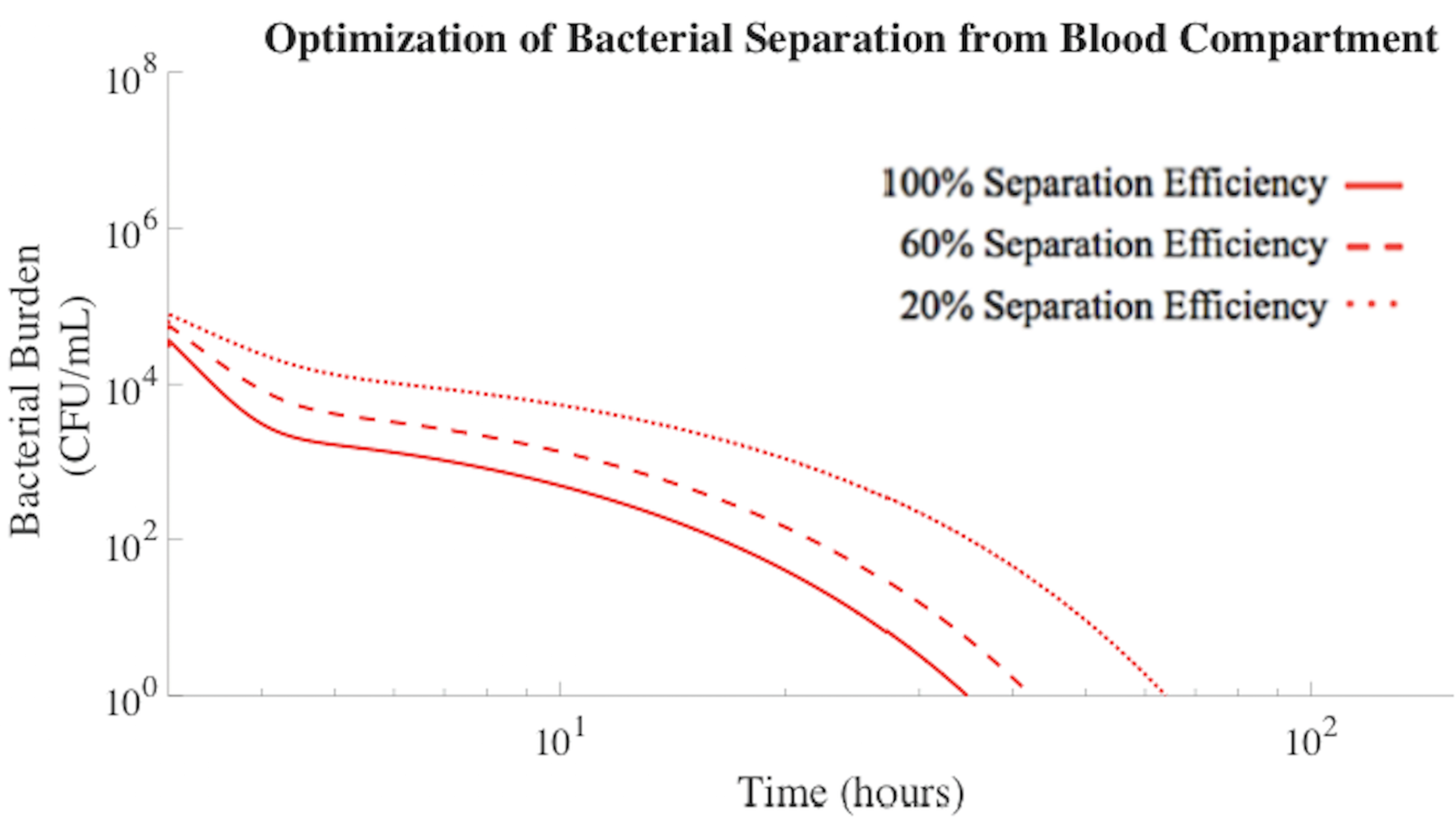

Supplement: S4 Fig — 20% bacterial separation efficiency was not efficient enough to significantly impact the overall bacterial clearance rate and resulted in the same clearance rates as antibiotic treatment alone. (TIFF) [file pone.0163167.s004.tiff]

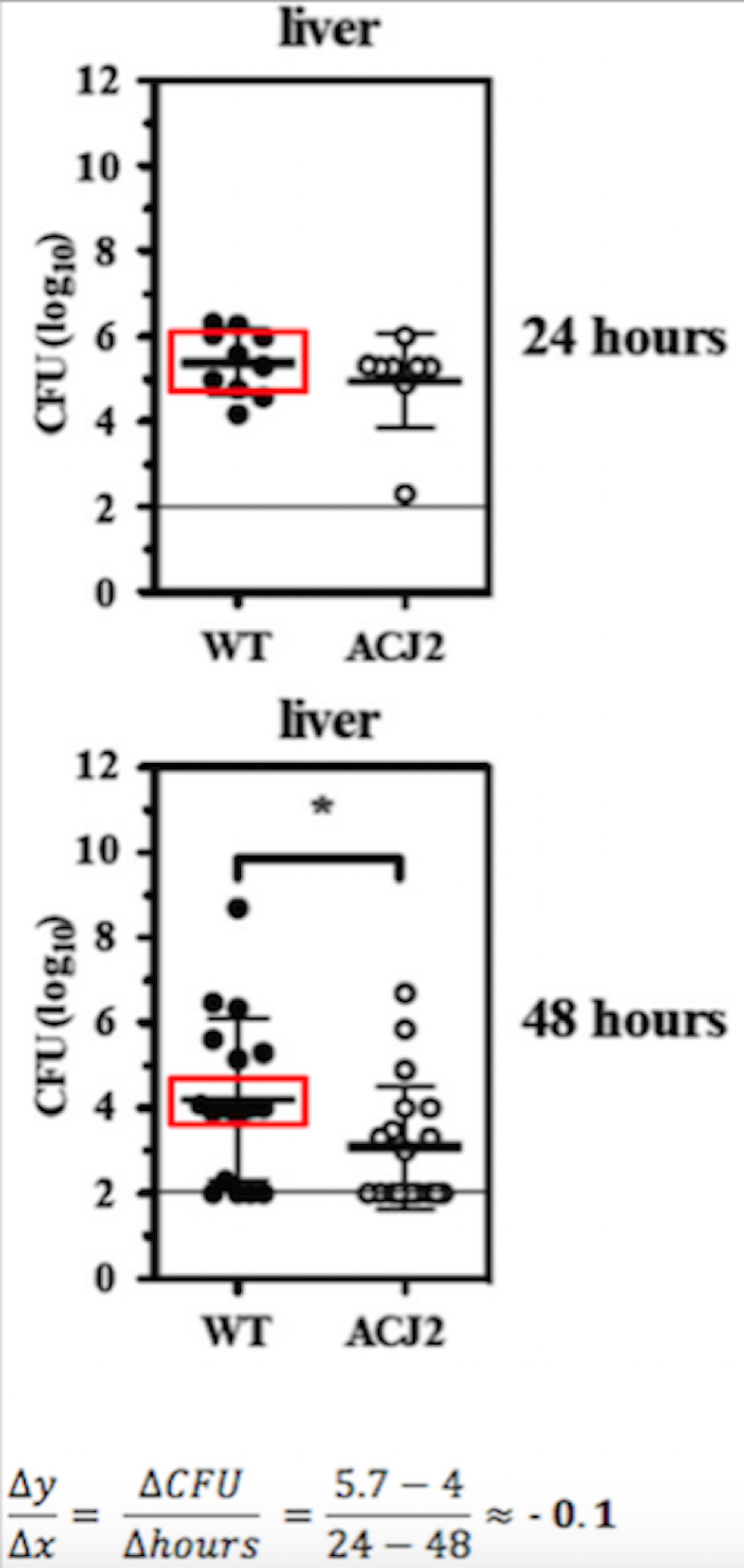

Supplement: S5 Fig — The net bacterial growth rate of A. baumannii in the liver of a non-immunocompromised rodent model is provided as an example calculation [21]. The linearization equation used to calculate all net bacterial growth rates is detailed in S5 Fig. (TIF) [file pone.0163167.s005.tif]

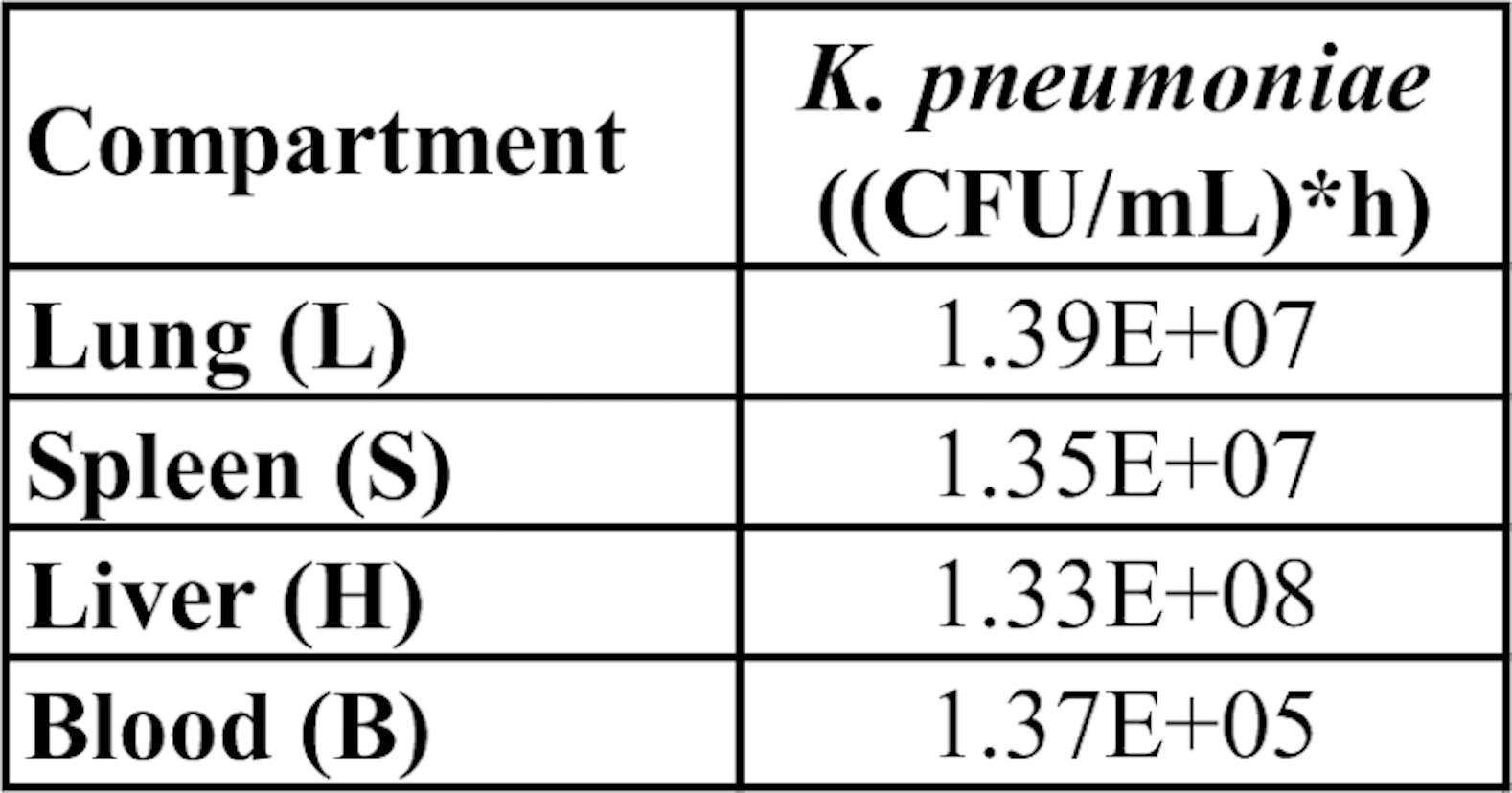

Supplement: S1 Table — (TIFF) [file pone.0163167.s006.tiff]

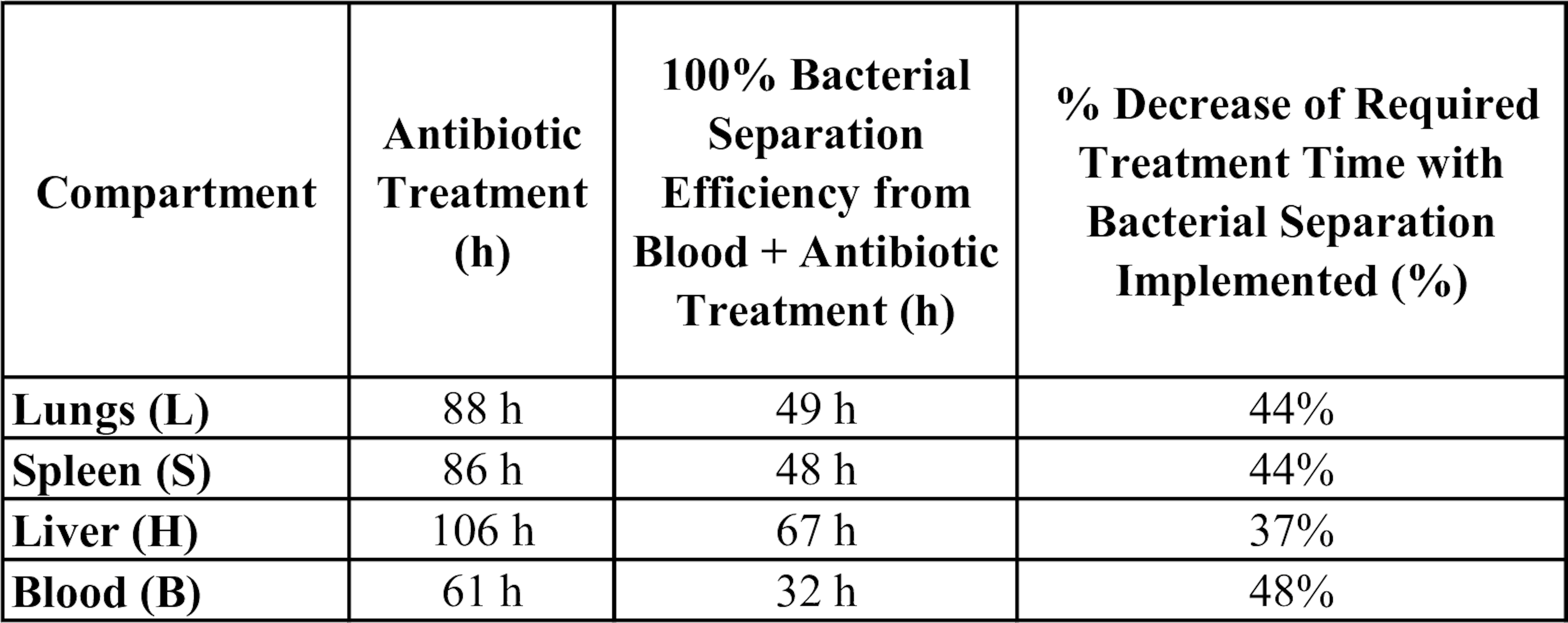

Supplement: S2 Table — (TIFF) [file pone.0163167.s007.tiff]

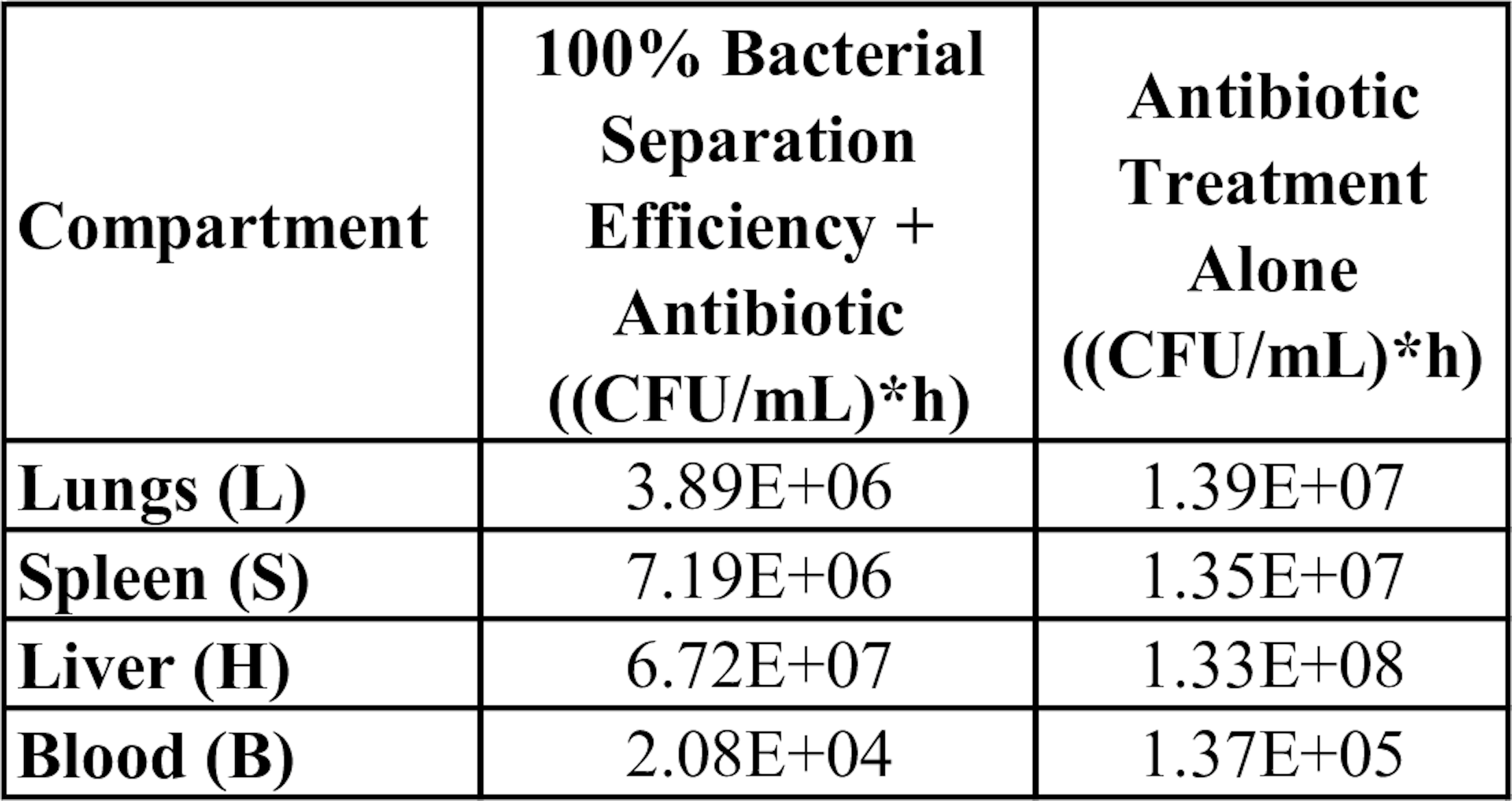

Supplement: S3 Table — (TIFF) [file pone.0163167.s008.tiff]

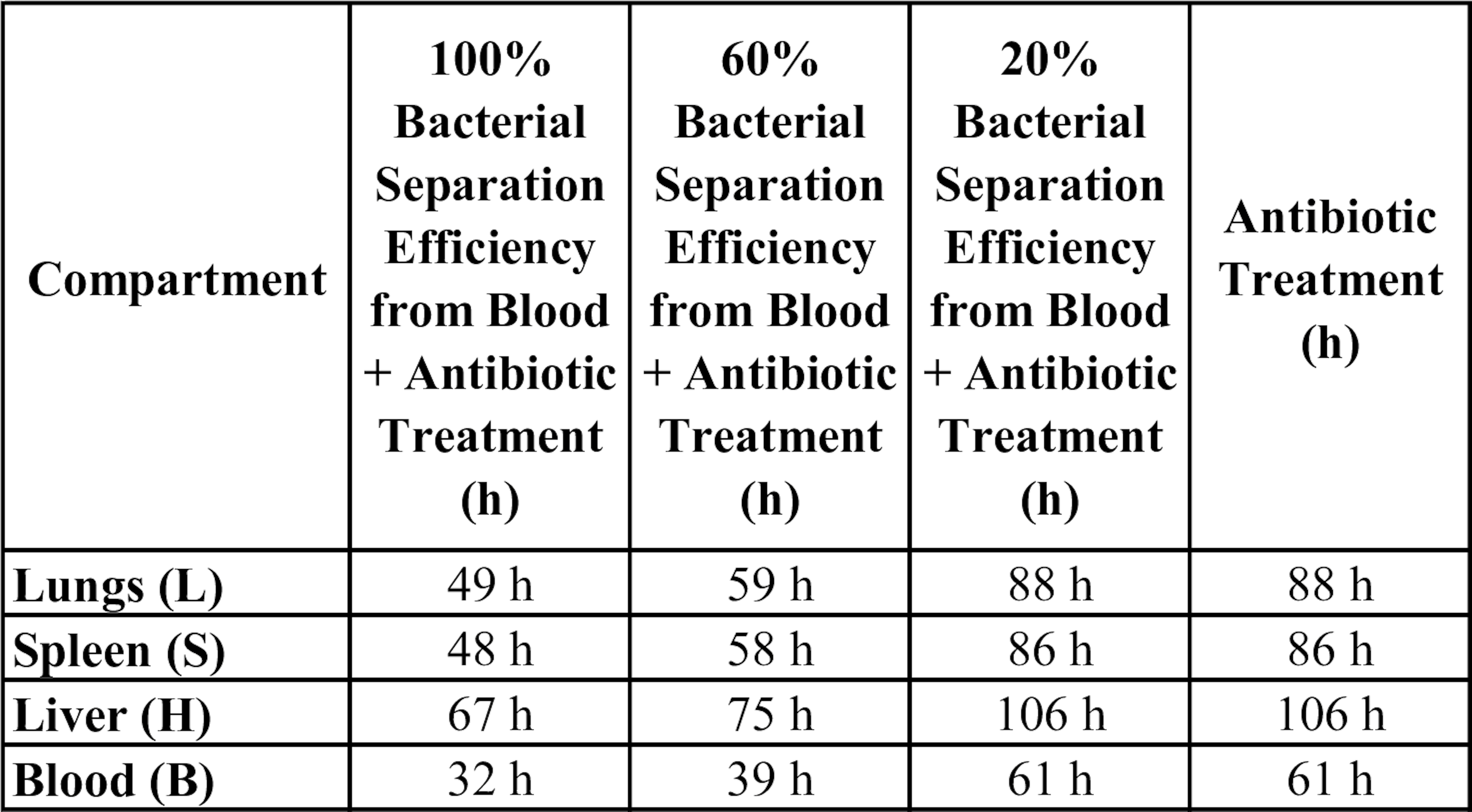

Supplement: S4 Table — (TIFF) [file pone.0163167.s009.tiff]

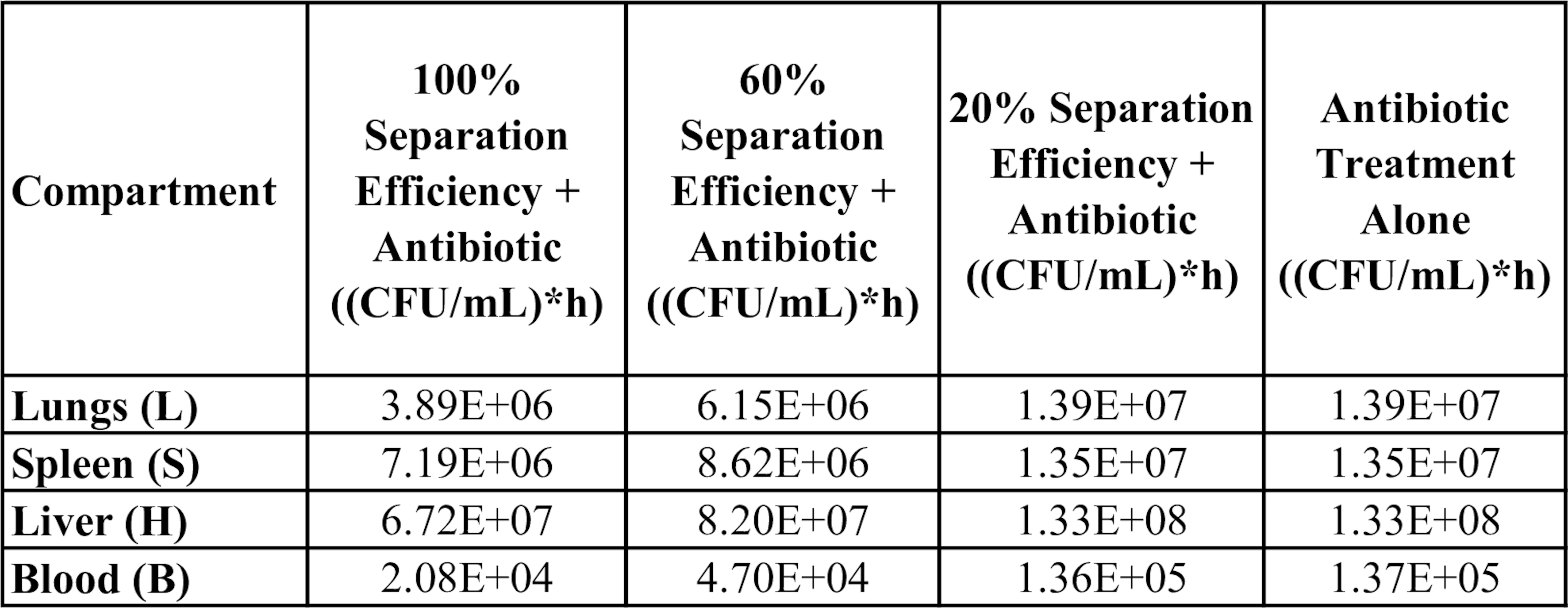

Supplement: S5 Table — (TIFF) [file pone.0163167.s010.tiff]
