# Supplementary material for: Dynamic Computational Model of Symptomatic Bacteremia to Inform Bacterial Separation Treatment Requirements
Source: PLoS One. 2016 Sep 22;11(9):e0163167. doi: 10.1371/journal.pone.0163167 (PMC5033423; doi:10.1371/journal.pone.0163167)
Supplement: S1 Text — (DOCX) [file pone.0163167.s011.docx]

function dydt = model(t,y)

dydt = zeros(size(y));

b_500 = 0.0025;

r_b = [25,30,43];

r_b = r_b .* 10^-9;

r_c = 6.5 * 10^-7;

b = b_500*(((5*10^-7)./r_b).^3);

k_B = 1.38*10^-23;

T = 300; %temperature in kelvin

n_b = 4 * (10^-3);

k_d_b = (2 * r_c * k_B * T)./(3 * n_b .* r_b);

r_t = 0.005;

Q_shear = 1/10;

gamma = (4 * Q_shear)/(pi * (r_t^3));

k_shear = pi * gamma .* (r_b + r_c).^3;

c_o_in = 10^7;

p = 0.5;

N = 4 * p * ((r_c./r_b).^2);

B = 40;

X = (4 * 3.5 * pi * (r_b.^3))/3;

u_o = (4 * pi) * 10^-7;

r_n = ((r_c.^3) + N .* (r_b.^3)).^(1/3);

v_mag_b = ((r_n.^2) .* N .* X .* B)./(9 * n_b * u_o);

Q = 1 * (2.778 * 10^-7);

a = 0.002 * 0.0006;

v_l = Q/(16 * a);

Length = 0.027;

t_res = Length/v_l;

L_h = 0.0006;

t_mag_b = (L_h./v_mag_b);

t_2 = linspace(10, 0, 156);

c_e_b = 3.7 * (10^-4);

c_o_b = (c_o_in) .* exp(-1 * c_e_b * (k_d_b + k_shear) .* b .* t_2);

if (t_res >= t_mag_b)

h_b = (1 - exp(-c_e_b * (k_d_b + k_shear) .* b .* c_o_b .* t_2)) .* 10^4;

elseif (t_res < t_mag_b)

h_b = (t_res .* ((1 - exp(-c_e_b * (k_d_b + k_shear) .* b .* c_o_b .* t_2))) .* 10^4) ./t_mag_b;

end

maxVal = max(h_b(:));

f_25 = (h_b(1,1)/maxVal);

f_30 = (h_b(1,2)/maxVal);

f_43 = (h_b(1,3)/maxVal);

%A. baumannii Growth Rate, Immunocompromised

%{

G_L = (0.21);

G_S = (0.14);

G_H = (0.10);

G_B = (0.08);

%}

%A. baumannii Growth Rate, Non-Immunocompromised

%{

G_L = (-1.74);

G_S = (-0.14);

G_H = (-0.10);

G_B = (-0.17);

%}

%A. baumannii Growth Rate, Antibiotic AND Immunocompromised

%{

G_L = -0.24;

G_S = -0.07;

G_H = -0.18;

G_B = -0.05;

%}

%K pneumoniae Growth Rate, Immunocompromised

%{

G_L = (0.10);

G_S = (0.11);

G_H = (0.13);

G_B = (0.15);

%}

%K pneumoniae Growth Rate, Non-Immunocompromised

%{

G_L = (-1.50);

G_S = (-0.11);

G_H = (-0.15);

G_B = (-0.10);

%}

%K pneumoniae Growth Rate, Antibiotic AND Immunocompromised

%{

G_L = (-0.35);

G_S = (-0.10);

G_H = (-0.15);

G_B = (0.02);

%}

%Volumes, Rodent

V_L = 2.10;

V_S = 0.15;

V_H = 1.99;

V_E = 0.002 * 0.0006 * Length * ((1 * 10^6)/1)

%Blood Flow Rates, Rodent

Q_L = (6.84) * 2.1;

Q_S = (1.2) * 0.15;

Q_H = (1.5) * 1.99;

Q_E = .36;

%Volumes, Human

V_L = 1315;

V_S = 127;

V_H = 830;

V_E = 90;

%Blood Flow Rates, Human

Q_L = 1.14 * 1315;

Q_S = 1.97 * 127;

Q_H = 0.97 * 830;

Q_E = 1000;

% partitioning coefficients

%{

p_L = 3;

p_S = 28;

p_H = 79;

p_E = 1;

%}

%{

p_L = 93;

p_S = 59;

p_H = 749;

p_E = 1;

%}

L = y(1);

S = y(2);

H = y(3);

E = y(4);

B = y(5);

G = y(6);

% NP Size

f = f_25;

f = f_30;

f = f_43;

dydt(1) = (G_L*L) + ((Q_L/V_L) * 60 * B) - ((Q_L/(V_L*p_L)) * 60 * L);

dydt(2) = (G_S*S) + ((Q_S/V_S) * 60 * B) - ((Q_S/(V_S*p_S)) * 60 * S);

dydt(3) = (G_H*H) + ((Q_H/V_H) * 60 * B) + ((Q_S/(V_S*p_S)) * 60 * S) - (((Q_H+Q_S)/(V_H*p_H)) * 60 * H);

dydt(4) = ((Q_E/(V_E)) * 60 .* B .* f) - ((Q_E/(p_E*V_E)) * 60 .* E .* (1 - f));

dydt(5) = (G_B*B) + (((Q_H+Q_S)/(V_H*p_H)) * 60 * H) + ((Q_L/(V_L*p_L)) * 60 * L) + ((Q_E/(p_E*V_E)) * 60 .* E .* (1 - f)) - (((Q_H/V_H) + (Q_S/V_S) + (Q_L/V_L) + (f * (Q_E/V_E))) * 60 * B);

dydt(6) = E;
